# *TERT* Promoter Mutated Follicular Thyroid Carcinomas Exhibit a Distinct microRNA Expressional Profile with Potential Implications for Tumor Progression

**DOI:** 10.1007/s12022-021-09695-w

**Published:** 2021-10-21

**Authors:** Johan O. Paulsson, Jan Zedenius, C. Christofer Juhlin

**Affiliations:** 1 Department of Oncology-Pathology, Karolinska Institutet, Stockholm, Sweden; 2 Department of Molecular Medicine and Surgery, Karolinska Institutet, Stockholm, Sweden; 3 Department of Breast, Endocrine Tumors and Sarcoma, Karolinska University Hospital, Stockholm, Sweden; 4 Department of Pathology and Cytology, Karolinska University Hospital, Stockholm, Sweden

**Keywords:** miRNA, TERT promoter, Mutation, Expression, Follicular thyroid carcinoma

The role of microRNAs (miRNAs) as regulators of various important biological processes in human tissues, including cancer development, is established. In thyroid cancer, subsets of miRNAs exhibit oncogenic or tumor suppressive functions, influencing diverse neoplastic processes such as tumor growth, apoptosis, invasive behavior, and survival [1]. Moreover, recurrent somatic mutations in the miRNA processor genes *DICER1* and *DGCR8* have been reported in subsets of well-differentiated thyroid carcinomas (WDTCs) and/or poorly differentiated thyroid carcinomas (PDTCs) [2–5], and altered levels of *DICER1* and *DGCR8* messenger RNA (mRNA) promote aberrant miRNA expression patterns in vitro and in vivo [2, 3, 5]. In all, these advances reinforce the notion that dysregulation of the miRNA landscape should be an important mechanism underlying thyroid tumor development and progression.

In terms of genetic events coupled to worse clinical prognosis in WDTC, recurrent somatic mutations of the *telomerase reverse transcriptase* (*TERT*) promoter region have been established as one of the most confident molecular markers of poor patient outcome [6, 7]. The mutations activate *TERT* gene output, enhancing immortalization through elongation of telomere repeats. *TERT* promoter mutations increase in frequency with more aggressive thyroid cancer subtypes and advanced stages of disease [6]. Apart from the well-established anti-senescence function of TERT, little is known regarding non-canonical effects on cellular function in thyroid cancer. We recently observed a specific global mRNA pattern in *TERT* promoter mutated follicular thyroid carcinomas (FTCs) compared to FTCs with *TERT* promoter wild-type sequences, and the former group was enriched in mRNAs associated to various metabolic pathways [3]. Intrigued by the notion that *TERT* promoter mutated tumors may exhibit a distinct molecular phenotype, we therefore sought to investigate the global miRNA expressional landscape in FTCs with known *TERT* promoter mutational status.

**Fig. 1:**
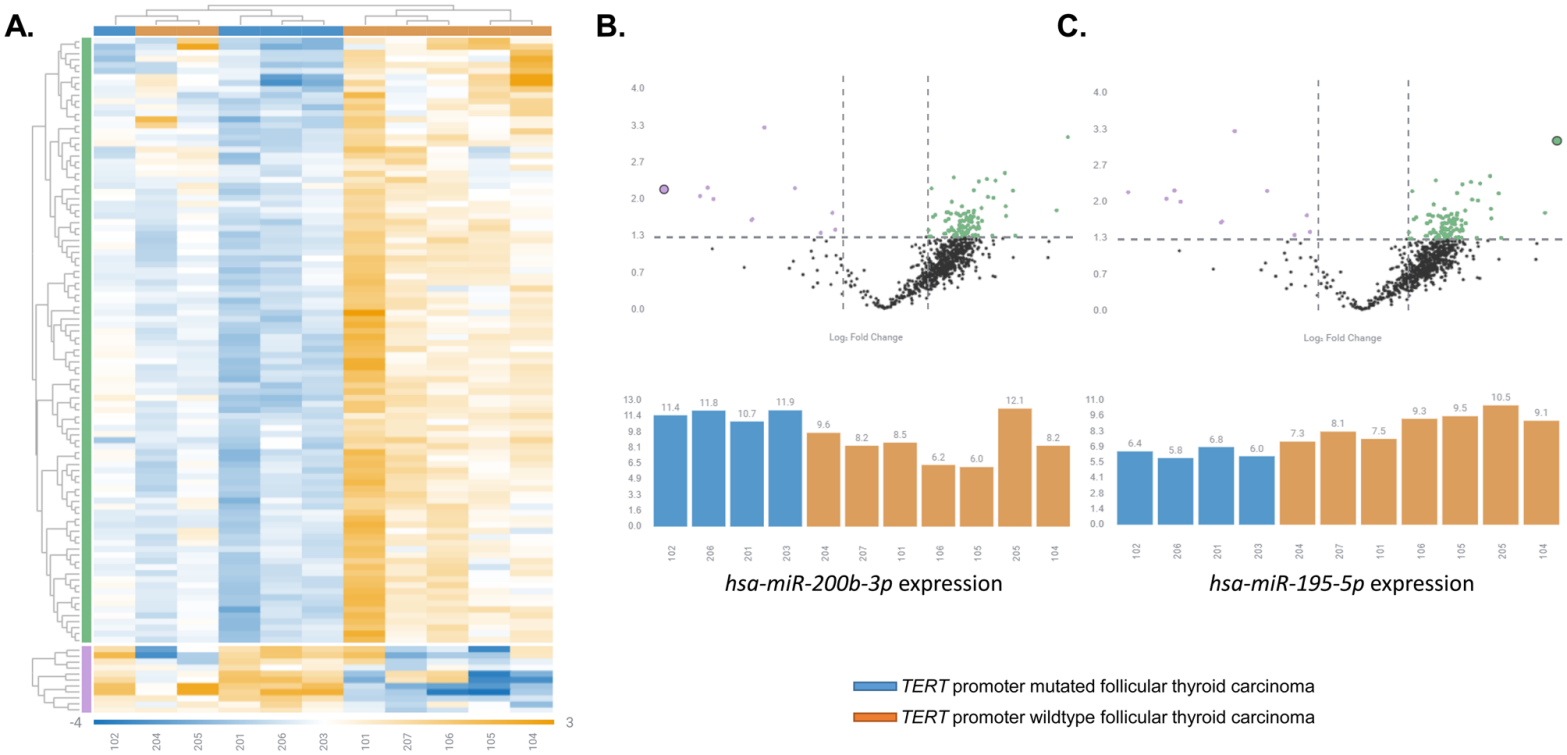
The distinct microRNA (miRNA) expressional profile in *TERT* promoter mutated follicular thyroid carcinoma (FTC). **A** Differential expression analysis of 827 miRNAs in FTC yielded 111 miRNAs with a significant expressional fold difference (≥ 1.5 or ≤ −1.5) with a *p* value < 0.05 in *TERT* promoter mutated FTCs marked in blue (*n*=4; cases 102, 201, 203, and 206) compared to wild-type cases marked in orange (*n*=7; cases 101, 104, 105, 106, 204, 205, and 207). In the heatmap, down-regulated miRNAs are represented in blue and up-regulated miRNAs in orange. **B** Volcano plot (top row) highlighting *hsa-miR-200b-3p* (largest purple dot), graph is shown with inverted logarithmic *p* values on the *Y*-axis and logarithmic fold change on the *X*-axis. Significantly up- and down-regulated miRNAs in the *TERT* promoter mutated FTCs are shown in the top left and right corner respectively. Below is the logarithmic normalized expression of *hsa-miR-200b-3p* in *TERT* promoter mutated FTCs (blue bars) compared to that of *TERT* promoter wild-type cases (orange bars). **C** Similar data shown for *hsa-miR-195-5p* (largest green dot in the volcano plot), the top down-regulated miRNA in the *TERT* promoter mutated group compared to *TERT* promoter wild-type tumors

We extrapolated data from a previously performed Nanostring nCounter platform analysis (Nanostring Technologies, Seattle, WA, USA) in which expression of 827 human miRNAs was interrogated in 11 FTCs with known *TERT* promoter genotypes [3]. Ethical approval was granted by the Swedish Ethical Review Authority (approval number 2015-959-31). The data was analyzed using Rosalind (Rosalind Inc. https://rosalind.onramp.bio/) as previously described [3]. Data normalization was performed in two steps, a positive control normalization and codeset normalization. During both steps, the geometric mean of each probeset was used to create a normalization factor. ROSALIND then calculates fold changes and *p* values for comparison using the *t*-test method and Benjamini-Hochberg method for estimating false discovery rates, assuming normal distribution. A differential expression analysis between *TERT* promoter mutated FTCs (*n*=4) and *TERT* promoter wild-type FTCs (*n*=7) was performed and visualized in a heatmap. Using a cutoff of ≥ 1.5 or ≤ −1.5 expressional fold change difference for up-regulated and down-regulated miRNAs respectively, and a *p* value set to <0.05, a total of 111 miRNA genes were found to be differentially expressed in *TERT* promoter mutated FTCs as compared to wild-type cases (Fig. 1A). The *TERT* promoter mutated cases displayed a high number of down-regulated miRNAs compared to the wild-type cases, and the top 5 most up- and down-regulated miRNAs in the former group are detailed in Table 1. The top down-regulated miR among *TERT* promoter mutated FTCs was hsa-miR-195-5p (fold change −5.73), and the top up-regulated miRNA was hsa-miR-200b-3p (fold change 8.25) (Table 1; Fig. 1B-C). Interestingly, hsa-miR-195-5p has been endowed with putative tumor suppressor functions in thyroid cancer, as it inhibits cell proliferation and invasion in thyroid cancer cell lines, while promoting apoptosis [8]. The most up-regulated miRNA in the *TERT* promoter mutated FTCs, hsa-miR-200b-3p, has been shown to be down-regulated in anaplastic thyroid carcinoma (ATC)—but little is known regarding the roles of this miRNA in WDTCs [9].

**Table 1:** Top 5 down- and up-regulated microRNAs in *TERT* promoter mutated follicular thyroid carcinomas compared to wild-type cases

**A.**	**Down-regulated miRNAs**		
	**miRNA**	**Fold change**	*p* value*
	*hsa-miR-195-5p*	−5.73173	0.00078
	*hsa-miR-145-5p*	−5.14378	0.01635
	*hsa-miR-143-3p*	−3.46785	0.04721
	*hsa-miR-497-5p*	−3.39323	0.00724
	*hsa-miR-519e-3p*	−3.17536	0.02091
**B.**	**Up-regulated miRNAs**		
	**miRNA**	**Fold change**	*p* value*
	*hsa-miR-200b-3p*	8.25061	0.00685
	*hsa-miR-200a-3p*	5.85009	0.00907
	*hsa-miR-222-3p*	5.43847	0.00640
	*hsa-miR-135a-5p*	5.14878	0.01027
	*hsa-miR-3151-5p*	3.57794	0.02489
**C.**	**Selected gene products with associations to cancer targeted by the differentially expressed miRNA pool**		
	**Gene**	**Function**	**#miRNAs targeting**
	*MDM2*	P53 regulator	34
	*CDK6*	Cell cycle regulator	31
	*DICER1*	miRNA regulator	30
	*CCND1*	Cell cycle regulator	29
	*CDKN1A*	Cell cycle regulator	29

^*^p values < 0.05 were considered statistically significant

In terms of gene products most frequently targeted by the differentially expressed miRNAs, the top 30 most affected validated genes included *MDM2*, *CDK6*, *DICER1*, *CCND1* (encoding cyclin D1), and *CDKN1A* (encoding p21), all exhibiting some association to the development or progression of thyroid cancer (Table 1). Using the Reactome Pathway Database (https://reactome.org/) for the top 30 most affected genes, the abovementioned observations were reinforced, with significant associations between these genes and the pathway terms “cell cycle,” “transcriptional regulation by TP53,” and “oncogene induced senescence.”

Two *TERT* promoter wild-type FTCs (cases 204 and 205) clustered together with the four *TERT* promoter mutated cases (Fig. 1), suggesting that other molecular mechanisms besides *TERT* promoter mutations establish the miRNA landscape of FTCs. A closer look at these two tumors in terms of *TERT* mRNA expression and alternative *TERT* gene aberrancies revealed neither *TERT* gene copy number gain, *TERT* promoter hypermethylation, or detectable *TERT* mRNA expression, all mechanisms previously described in FTCs [10]. Thus, other contributing mechanisms to the observed miRNA pattern are likely to occur in these two cases.

In this pilot series, we demonstrate that *TERT* promoter mutated FTCs carry a unique miRNA signature and highlight a number of interesting candidates for future studies in terms of disease progression and prognosis. The observed down-regulation of hsa-miR-195-5p should be of particular interest for future studies, as this miRNA could constitute a potent tumor suppressor in thyroid cancer cell lines by targeting *TERT* mRNA [8]. Moreover, cancer-related mRNA targets of the differentially expressed miRNAs were amassed in cell cycle and P53 signaling, providing a possible link between *TERT* promoter mutations and dysregulation of these pathways in FTCs. Interestingly, *DICER1* was also found among the top targeted mRNAs in this dataset, which may suggest a complex loop in which the mRNA translation of this miRNA processor itself is affected by differentially expressed miRNAs in *TERT* promoter mutated FTCs. However, the results obtained from this study are based on a small sample series in need of experimental validation. For example, our findings of overexpressed hsa-miR-200b-3p in *TERT* promoter mutated FTCs are in conflict with the previously demonstrated down-regulation in ATCs, thereby serving as a good example for a miRNA candidate reliant on further analyses using larger tumor cohorts as well as the inclusion of normal controls [9].

In conclusion, *TERT* promoter mutated FTCs may exhibit a specific miRNA pattern that in part regulates key cancer pathways, an observation that may support non-canonical *TERT* functions in thyroid cancer. Given the dismal outcome for many *TERT* promoter mutated FTCs, increased knowledge of the molecular underpinnings driving these tumors should be of clinical interest.
